# Diode laser in management of loss of taste sensation in patients with post-COVID syndrome: a randomized clinical trial

**DOI:** 10.1186/s12903-023-02952-w

**Published:** 2023-05-06

**Authors:** Alshaimaa Ahmed Shabaan, Islam Kassem, Aliaa Ibrahium Mahrous, Inass aboulmagd, Mai Badrah, Mohamed Attalla, Shaimaa Mohsen Refahee

**Affiliations:** 1 Oral & Maxillofacial Surgery Department, Faculty of Dentistry, Fayoum University, Fayoum, Egypt; 2 Department of Maxillofacial Surgery, Al-Alamin Hospital, Ministry of Health, Al-Alamin, Egypt; 3 Fixed Prosthodontic Department, Faculty of Dentistry, Fayoum University, Fayoum, Egypt; 4 Fixed Prosthodontic Department, Faculty of Dentistry, October University for Modern Sciences and Arts, Giza, Egypt; 5 Oral & Maxillofacial Radiology Department, Faculty of Dentistry, Fayoum University, Fayoum, Egypt; 6 Internal Medicine, Faculty of Medicine, Alexandria University, Alexandria, Egypt; 7 Oral and Maxillofacial Department, IMAXFAX, Alexandria, Egypt

**Keywords:** COVID-19, Taste disorder, Ageusia, Laser therapy, Low-level, Diode laser, Recovery of function, Quality of life

## Abstract

**Objective:**

Loss of taste (ageusia) is a symptom observed following recovery from COVID-19 infection. The loss of taste and smell sensation may negatively affect patients’ quality of life (QoL). The present study aimed to evaluate the effectiveness of the Diode Laser in managing loss of taste sensation in patients with post-COVID syndrome versus the placebo.

**Material and method:**

The study sample was 36 patients who complained of persistent loss of taste sensation following COVID-19. The patients were randomly assigned to one of the two groups according to the received treatment: Group I (laser treatment) and Group II (light treatment), with each patient receiving a diode laser treatment or placebo from the same operator. Taste sensation was subjectively measured after treatment for four weeks.

**Results:**

The results demonstrated a significant difference between both groups regarding taste restoration after one month (p = 0.041), with Group II having a significantly higher percentage of cases 7 (38.9%) with partial taste restoration. In contrast, a significantly higher proportion of Group I 17 cases (94.4%) had complete taste restoration (p < 0.001).

**Conclusion:**

The present study concluded that using a Diode laser 810 nm aided in a more rapid recovery from loss of taste dysfunction.

## Introduction

Severe acute respiratory syndrome coronavirus 2 (SARS-CoV 2) infection lead to a global pandemic with a significant impact on health, social aspects, and wealth. Since the breakout of the virus in china, 80% of infected patients have developed mild to moderate symptoms, while 5% have developed severe symptoms. The virus mortality rate ranged between 3 and 12% of infected patients [1–4].

Various studies reported a wide range of symptoms, such as fever, dry cough, and, in some cases, shortness of breath, dysosmia, and dysgeusia. In addition to these symptoms, many authors reported different oral manifestations in patients with SARS- Cov2 infection. These manifestations could range from self-limiting disorders like unspecific oral ulceration, desquamative gingivitis, petechiae, and co-infection like candidiasis to more complicated manifestations like mucormycosis [1–7].

Loss of taste sensation was one of the early or subclinical symptoms of SARS-CoV2. The prevalence ranged from 47 to 67% in patients with SARS-CoV2 infection, and 20% of all patients reported it as an isolated or as the first symptom [8].

Those signs and symptoms of SARS-CoV2 infection lasted 7–10 days in mild to moderate cases; recovery can take up to six months in severe cases. Many studies report that a large percentage of patients who recovered from SARS-CoV 2 infection may have one or more symptoms that last for weeks or even months. These long-lasting symptoms were referred to as post-COVID syndrome or long COVID-19. The prevalence of long COVID ranged between 10 − 47% and could persist for 8–12 weeks [1, 9].

Loss of taste sensation is one of the symptoms that had a high prevalence among patients with long COVID. Moraschini et al. [10] concluded that 18.8% and 14.1% of patients who recovered from COVID-19 still had persistent symptoms long-term as anosmia and ageusia, respectively, after a follow-up period 67 days. Different studies reported the impact of QoL, including decreased pleasure in food, poor appetite, trouble with cooking and detecting spoiled food, alteration in body weight, feelings of vulnerability, mood changes, depression, and deterioration in social interactions [11, 12].

Different medications have been used to treat post-COVID loss of taste, including oral corticosteroids, zinc sulfate, topical corticosteroids, theophylline, caroverine, vitamin A, sodium citrate, and minocycline. However, scientific evidence on the effectiveness of these drugs was limited, and no randomized controlled trials exist [13, 14].

The current study has revealed that the pathogenicity of SARS-Cov2 was initiated by the virus’s targeting of ACE-2 receptors, which allow viral entry into cells. ACE-2 receptors are found throughout the oral mucosa, particularly on the tongue and in the salivary glands. With viral-induced hyperinflammation, the downregulation of ACE-2 receptors may result in endothelial dysfunction, as well as dysfunction at viral entry points and in multiple organs. This chain of events may reveal a possible mechanism for loss of taste by demonstrating the unique mechanisms and interactions between thrombosis and inflammation [15].

This study proposes the diode laser 940 as a possible treatment for the loss of taste sensation in patients with long COVID. The low-level laser using diode lasers of wavelengths 588–940 nm had bio-stimulatory impacts on target tissues. In addition, it regulates biochemical processes by improving the anti-oxidant system that can boost mitochondria breathing, reduce tissue damage and produce ATP [16]. The present study aimed to evaluate the effectiveness of the Diode Laser 940 nm in managing loss of taste sensation in patients with post-COVID syndrome versus the placebo.

## Materials and methods

The study was conducted from May 2021 to August 2021 at the Oral and Maxillofacial Department, Faculty of Dentistry, Fayoum University. The Study protocol was registered in ClinicalTrials.org (NCT04821999) and approved by the Research Ethics Committee under the approval code EC 2130. The study procedures were conducted in accordance with the Declaration of Helsinki and CONSORT guidelines 2012 [17].

### Study design

The study was a prospective, randomized (1:1), blinded clinical trial. The randomization ensures that an equal number of participants are assigned to each group using a random block design with blocks of sizes 2, 4, and 6 within each group. Patients were not aware of the type of treatment they were receiving. After recovery from COVID-19, 36 patients in the study complained of persistent loss of taste sensation. The selection of patients was determined by inclusion and exclusion criteria. The inclusion criteria include Adults (> 18 years old) with a positive test for SARS CoV- 2 via reverse transcription polymerase chain reaction (RT-PCR). After recovering from the COVID-19 infection, patients continued to experience a persistent loss of 4 basic taste sensations for at least four weeks after COVID-19 recovery. Patients with pre-existing olfactory or gustatory dysfunction without a laboratory-confirmed COVID-19 infection diagnosis, pregnant women, patients with any systemic disease, patients who received any treatment to accelerate gustatory function recovery, or taking any medications that cause loss of taste or smell were excluded from the study.

**Fig. 1:**
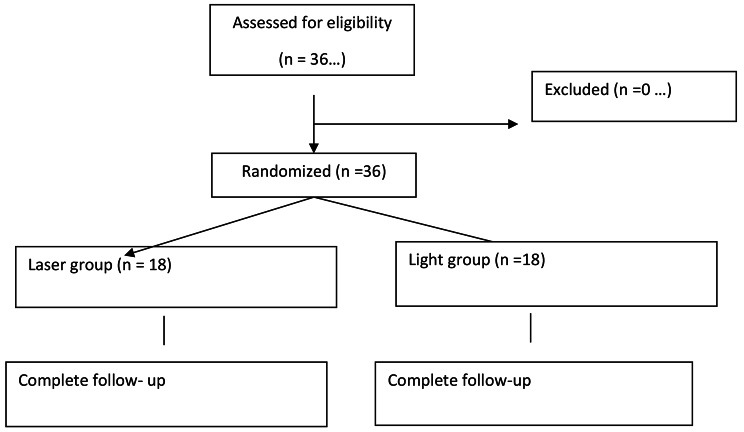
Consort statement flow chart

The patients were randomly assigned (1:1) to one of the two groups according to the treatment they received: Group I (laser treatment), where each patient received a diode laser treatment, and Group II (light treatment), where each patient was received placebo by the same operator (Fig. 1).

Demographic data were recorded for all the patients: age, gender, previous clinical history, and swab positivity. COVID-19 signs and symptoms were detected in patients’ clinical records. The patients were interviewed to obtain a detailed timeline of loss of taste sensation regarding the onset and severity. A standardized and validated test was performed preoperatively to evaluate the gustatory function. The test investigates the ability to perceive four primary tastes (sweet, salty, sour, and bitter). The primary predictor variable was the treatment method used. The patients and assessors were blinded throughout the period of the study.

### Sample size estimation

The primary outcome measure was the recovery of taste sensation. A sample calculation (STATA V16.0) performed using data from a previous study showed that a total of 32 patients would need to be included for a study power of 80% at an alpha of 0.05. However, this study included a total of 36 patients (18 in each group) to account for any possible dropouts [18].

### Intervention

All patients were treated by the same practitioner. Before the treatment, both the patient and clinician donned protective eyewear.

In Group I, the patient received laser therapy using a 940-nm diode laser (EPIC^TM^, BIOLASE, www.biolase.com) with an adjustable handpiece that created a spot size of 15 mm. The power output was set at 1 W continuous wave. The handpiece was moved over the tongue for two 3-minute intervals separated by a 30-second rest period. The total time of the laser was 6 min on tissue. In Group II, the same procedures were followed except that patients received a light emitted from the therapy handpiece to act as a placebo.

### Outcome measurement

A standardized and validated test was used to evaluate the gustatory function. The test aimed to investigate patients’ ability to perceive four primary tastes (sweet, salty, sour, and bitter) [19].

The test used four solutions prepared by the assessor to test the four primary tastes. The solutions were prepared as follows: 30 g of table salt was added to 1 L of deionized water for the salted solution, a sweet solution prepared as 30 g of refined sugar was dissolved in 1 L of deionized water, Sour solution was prepared as 90 mL of commercial 100% lemon juice added to 1 L of deionized water and unsweetened decaffeinated coffee act as the Bitter solution.

During the test, each patient received 1 mL of each solution dropped in the center of the tongue. The patient was indicated whether the perceived flavor was sweet, salty, bitter, or acid. Deionized water was used as a mouthwash between the different solution tests to wash any excess and avoid any mix between tested solutions. The solutions were presented in random order. In contrast, the bitter taste was presented last as it altered subsequent taste perception. The answers were recorded as either yes or no taste for each solution. The patient was recorded as totally recovered if they could perceive all the primary tastes. The patient was considered partially recovered if they failed to perceive one of the primary tastes.

The taste was assessed at the following intervals: during diagnosis, 1-week, 2-weeks, 3- week and 4 weeks post-treatment.

### Statistical analysis

Categorical data were presented as frequency and percentage values and were analyzed using Fisher’s exact test, followed by pairwise comparisons utilizing multiple z-tests with Bonferroni correction. Numerical data were analyzed for normality using Shapiro-Wilk’s test. Data showed parametric distribution, so they were presented as mean and standard deviation values and were analyzed using the independent t-test. The significance level was set at p < 0.05 within all tests. Statistical analysis was performed with R statistical analysis software version 4.1.1 for Windows1.

## Results

This study included 36 patients who were equally allocated into two groups according to the treatment modality (18 patients each). There was 3(16.7%) males and 15(83.3%) females in group I, while there was 5(27.8%) males and 13(72.2%) in the Group II. The mean age of the cases in Group I was (38.22 ± 12.90) years, whereas, in Group II, it was (44.78 ± 16.67) years. There was no significant difference between both groups regarding gender and age (p > 0.05). All patients were analyzed, and a general description of all clinical features, comorbidities, and treatment received was recorded. (Table 1)

**Table 1:** Intergroup comparison of demographic and clinical data

Parameter		Group I	Group II	P-value
Sex	Male	3 (16.7%)	5(27.8%)	**0.691**
	Female	15 (83.3%)	13 (72.2%)	
Age (years) Mean ± SD		38.22 ± 12.90	44.78 ± 16.67	0.196
***Co-moralities***	No	12 (66.7%)	9 (50.0%)	**0.500**
	Yes	6 (33.3%)	9 (50.0%)	
***Associated symptoms***	Asymptomatic	10(55.6%)	13(72.2%)	**0.246**
	Symptomatic	8(44.4%)	5(27.7%)	
Smoking History	Current smokers	6 (33.3%)	5 (27.7%)	0.153
	Non smokers	12(66.6%)	13(72.2%)	
Hospitalized	No	15 (83.3%)	13 (72.2%)	0.691
	Yes	3 (16.7%)	5(27.8%)	

Before treatment, taste sensation features were reported regarding loss of taste of sensation at diagnosis, the time elapsed since recovery (month), and associated Olfactory dysfunction. Ten patients in Group I (55%) reported a loss of taste sensation at the time of COVID-19 diagnosis, while in Group II, 13 patients reported a loss of taste at the time of diagnosis. In Group I and Group II, the time since recovery was 1.17 ± 0.53 and 0.93 ± 0.28 months, respectively. Six patients in Group I (33.3%) reported olfactory dysfunction associated with taste dysfunction, while nine patients in Group II reported olfactory dysfunction. There was no significant difference between both groups for all these parameters.

**Fig. 2:**
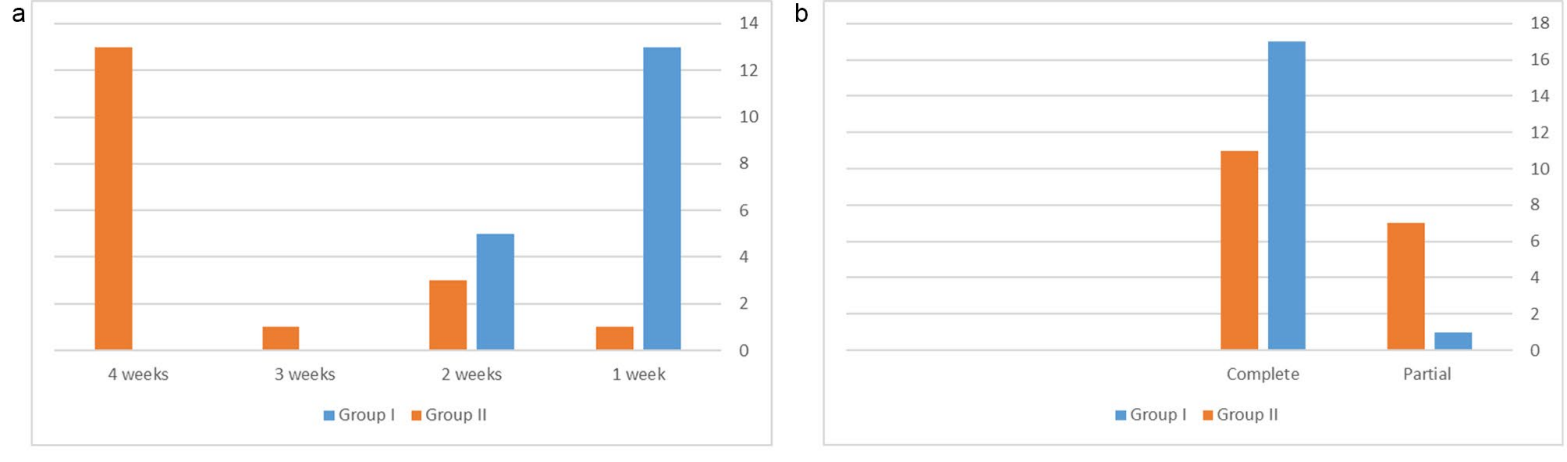
Intergroup comparison of outcome parameters 2-a Patient restore taste after 1week, 2,3,4 weeks 2-b: Taste restored after one month complete or partial

There was a significant difference between both groups regarding the time elapsed until taste restoration (p < 0.001). Post hoc pairwise comparisons showed Group I to have a significantly higher percentage of cases 13(72.2%) restoring taste after one week. In addition, they showed Group II to have a significantly higher percentage of cases 13(72.2%) restoring taste after four weeks (p < 0.001). Similarly, there was a significant difference between both groups regarding the status of taste restored after one month (p = 0.041), with Group II having a significantly higher percentage of cases 7(38.9%) partially restoring taste, while Group I having a significantly higher percentage of cases 17(94.4%) having complete restoration of taste (p < 0.001). (Fig. 2a,b)

## Discussion

Despite the development of a vaccine that helped contain the pandemic, healthcare providers still face the ongoing spread of SARS-Cov-2 globally with unprecedented COVID-19-related symptoms and morbidity. Many reports have documented a high incidence of taste and smell Sensation dysfunctions in COVID-19 patients. Loss of taste (ageusia) is a symptom observed following recovery from COVID-19 infection [1–3]. A Population-based study found that, in non-hospitalized subjects contacted for reporting persistent symptoms, 65% reported a loss of smell and 69% loss of taste at diagnosis, and 12% reported the loss of smell and 10% loss of taste a median of 117 days from disease diagnosis [20]. As the loss of taste sensation presents Unfavorable effects on patients’ QoL, many therapeutic strategies have been reported to help the patient recover from the loss of taste sensation [21–23].

Low-level lasers have several applications in dentistry. It has been used to enhance wound healing, gingival depigmentation, acceleration of tissue regeneration, treatment of temporomandibular disorders, alleviation of chronic orofacial pain, and induction of bone regeneration. Numerous studies have demonstrated that 940 to 980 nm diode laser wavelengths effectively reduce postoperative inflammation, enhance wound healing, and accelerate tissue regeneration.This study presents the diode laser 940 as a potential treatment for the loss of taste sensation in COVID patients. The diode laser 940 had an energy density of 4 J/cm2, which is considered the minimal energy density necessary for postoperative pain relief, anti-inflammatory effect, and edema elimination [24–26].

This study included 36 patients with long COVID that received either diode laser 940 or a placebo. The proposed treatment strategy brought a positive consequence to the resolution of loss of taste sensation as their results showed that patients treated with diode laser 940 have a significantly higher percentage of cases 13 (72.2%) restoring taste after one week. Moreover, patients treated with diode laser 940 have a significantly higher percentage of cases 17(94.4%) having complete restoration of taste.

The patients in the present study had persistent symptoms for a mean of 1.17 ± 0.53 months in Group I and 0.93 ± 0.28 months in Group II. This could be considered a relatively short period for long COVID, lasting from 8 to 12 weeks [1, 9].

Moraschini et al. [10] concluded that 18.8% and 14.1% of patients who recovered from COVID-19 still have persistent symptoms, long-term as anosmia and ageusia, respectively, after a follow-up period of 67 days. This short period of long COVID may be contributed to the impact on QoL. Brasseler et al. [23] investigate the incidence of subsequent eating disorders in these patients and SARS-CoV-2 positive patients who did not experience anosmia and ageusia during the same period and concluded that 6 out of 24 patients demonstrated increased fixation on their eating behavior post-COVID and over time these patients developed anorexia nervosa.

The present study showed the resolution of loss of taste sensation as their results showed that patients treated with diode laser 940 have a significantly higher percentage of cases 13(72.2%) restoring taste after one week. Rochmawati and Kamilah [27] proposed that the persistence of symptoms in COVID-19 patients was attributed to the overexpression of mitochondrial proteins and the presence of inflammatory markers. In addition, they found increased pro-inflammatory cytokines that could contribute to cytokine dysregulation and a cytokine storm.

The use of low level laser improved cellular metabolism by stimulating photoreceptors in the mitochondrial respiratory chain, releasing growth factors, and changing cellular ATP levels [28]. Fahimipour et al. [29] suggested that LLLT inhibits severe inflammatory reactions and promotes collagen formation. Qadri et al. [30] demonstrated that low-level lasers reduce periodontal and gingival inflammation regardless of wavelength. Moreover, the low-level laser with wavelengths ranging up to 970 nm improved the activity and number of T lymphocytes that produce cytokines responsible for tissue regeneration. Therefore, improved lymphocyte activity stimulates the phagocytic activity of macrophages, which is essential for the healing process. The low-level laser accelerates tissue regeneration and activation of neoangiogenesis, increasing tissue oxygen pressure [31–35]. The present study used single-session low-level therapy in accordance with Momeni et al., who assessed the effect of extraoral 940 nm low-level diode laser on pain, edema, and trismus following surgical extraction of impacted mandibular third molars. They concluded that Single-session irradiation of a 940 nm diode laser could effectively decrease pain following third molar extraction surgery. They claimed that multiple laser therapy sessions are time-consuming for both patients and clinicians and often decrease patient compliance.

The present study had several limitations. The present study had a mean young age of participants that limited the study results to the young age group, as age had a significant role in recovery from chemosensory deficits. Moreover, the small sample size did not give enough power to show differences in other demographic data, such as smoking and patients’ comorbidities [36].

Despite the mentioned limitations, analyzing patients’ chemosensory disorders through objective tests is a more effective technique for follow-up patients with chemosensory disturbances and avoiding possible under-or over-reporting biases.

## Conclusion

The findings of this study indicate that low-level laser therapy is effective in restoring taste sensation in patients with post-COVID-19 dysfunction. Further studies with improved design could address the study limitation and evaluate the current treatment modality in conditions other than SARS-Cov2 patients.

## Data Availability

The data supporting this study’s findings are available from the Faculty of Dentistry and Fayoum for health insurance hospitals. However, restrictions apply to the availability of these data, which were used under license for the current study and are not publicly available.
